# Morphological Similarity and Ecological Overlap in Two Rotifer Species

**DOI:** 10.1371/journal.pone.0057087

**Published:** 2013-02-22

**Authors:** Carmen Gabaldón, Javier Montero-Pau, Manuel Serra, María José Carmona

**Affiliations:** Institut Cavanilles de Biodiversitat i Biologia Evolutiva, Universitat de València, Valencia, Spain; Consiglio Nazionale delle Ricerche (CNR), Italy

## Abstract

Co-occurrence of cryptic species raises theoretically relevant questions regarding their coexistence and ecological similarity. Given their great morphological similitude and close phylogenetic relationship (i.e., niche retention), these species will have similar ecological requirements and are expected to have strong competitive interactions. This raises the problem of finding the mechanisms that may explain the coexistence of cryptic species and challenges the conventional view of coexistence based on niche differentiation. The cryptic species complex of the rotifer *Brachionus plicatilis* is an excellent model to study these questions and to test hypotheses regarding ecological differentiation. Rotifer species within this complex are filtering zooplankters commonly found inhabiting the same ponds across the Iberian Peninsula and exhibit an extremely similar morphology—some of them being even virtually identical. Here, we explore whether subtle differences in body size and morphology translate into ecological differentiation by comparing two extremely morphologically similar species belonging to this complex: *B. plicatilis* and *B. manjavacas*. We focus on three key ecological features related to body size: (1) functional response, expressed by clearance rates; (2) tolerance to starvation, measured by growth and reproduction; and (3) vulnerability to copepod predation, measured by the number of preyed upon neonates. No major differences between *B. plicatilis* and *B. manjavacas* were found in the response to these features. Our results demonstrate the existence of a substantial niche overlap, suggesting that the subtle size differences between these two cryptic species are not sufficient to explain their coexistence. This lack of evidence for ecological differentiation in the studied biotic niche features is in agreement with the phylogenetic limiting similarity hypothesis but requires a mechanistic explanation of the coexistence of these species not based on differentiation related to biotic niche axes.

## Introduction

In the last decade, molecular approaches have revealed great biological diversity in the form of cryptic species [1]–[3]. Co-occurrence of these species is common [4] and raises important questions, especially in terms of their coexistence and ecological similarity. Given their great morphological similitude and close phylogenetic relationship, these species are expected to have similar environmental requirements (i.e., niche retention) [5]–[7] and thus strong competitive interactions. Consequently, cryptic species are expected to be prone to competitive exclusion (i.e., the limiting similarity principle) [8]. The apparent lack of phenotypic and/or ecological differences between cryptic species raises the problem of finding the processes that may explain their co-occurrence and challenges the conventional view of coexistence [9], [10] because species coexistence has traditionally been explained by niche differentiation mechanisms (e.g., partitioning of resources, differential risk to enemies, temporal and spatial patchiness, and environmental fluctuations). However, there are alternative processes not based on biotic niche axis differentiation that could explain the co-occurrence of ecologically similar species, such as density-dependent life-history adjustments [11], [12] or those invoked by neutral models [13]. In cryptic species, this differentiation either does not exist or is subtle. Although the degree of ecological differentiation needed for stable coexistence depends on the degree of fitness differences (i.e., the more similar their fitness, the less difference is required) [14], it is still unclear how subtle these ecological differences that promote coexistence can be. Thus, the study of co-occurring cryptic species can illuminate the existing mechanisms, aid in the discovery of new ones, and offer the opportunity to experimentally quantify concepts such as limiting similarity.

Body size affects life history, the ecological niche of an organism, and its interactions with other organisms. The impact of body size in determining the ecological niche is especially significant in aquatic systems [15], as body size has implications for predation susceptibility and competitive ability. Many aquatic invertebrate predators detect their prey by mechanoreception [16], [17]; thus, the greater the size of the prey, the greater the mechanical disturbance created, and the greater the risk of being detected [18]. Additionally, aquatic vertebrate predators such as fishes use visual orientation to capture prey so that the reactive distance of the predator is positively related to the prey size [19]–[22]. Body size also shapes the consumer niche because large prey is more difficult to catch and has longer handling times [18], [23]–[25] or, in the case of filter-feeding zooplankton, because the size limit of the comestible particles is determined by the mesh width of the filtering apparatus [26]. Thus, a higher similarity in predation vulnerability and consumer niche is expected among competing species with similar body size and morphology.

The objective of this study is to explore whether subtle differences in body size and morphology translate into ecological differentiation. To this end, we used two cryptic rotifers species belonging to the *Brachionus plicatilis* species complex [1], *B. plicatilis* and *B. manjavacas*, as a model. Both species are commonly found living in sympatry in the plankton of many bodies of salt water in the Iberian Peninsula [27], [28]. The rotifer communities of these habitats are poorly diversified, and populations are expected to be regulated by food availability and predation [29]. The ponds inhabited by *B. plicatilis* and *B. manjavacas* are shallow, with low spatial heterogeneity but a highly variable salinity regime [30], and it has been suggested that a differential response to salinity could mediate the stable coexistence of the rotifers [31]. Both species are virtually morphologically identical. The only reliable feature for morphological identification is the shape of small accessory pieces of the internal masticatory apparatus (i.e., satellites) [32]. In addition, *B. plicatilis* is on average 6% longer than *B. manjavacas* [33]. In this study, we focus on three key ecological features (vulnerability to predation, food particle size preference, and starvation tolerance) where body size has been proven to be determinant in the *Brachionus* genus.

Species of the *B. plicatilis* complex lack any conspicuous escape response from predators or structures of protection such as spines, apart from the lorica, a hard outer covering of chitin [34]; thus, body size is a crucial factor in their susceptibility to predation by copepods [35], [36], [25]. Rotifers are primarily passive filterers, and their diets are affected by the structure and size of their feeding structures. For example, when comparing the maximum size of particles ingested by a *B. plicatilis* species, Hino and Hirano [37] concluded that the largest particle size that a rotifer is able to capture is dependent on its body size. This is most likely because the food groove, which is responsible for transporting the collected particles to the mouth using cilia, increases with body size, as has been demonstrated for gastropod larvae [38]. Interestingly, the body size of some species of *B. plicatilis* is clearly related to both the optimal particle size and the width of the retention spectrum [39]. In addition, for some rotifer species, feeding efficiencies increased with increasing dietary particle size [40], [41], although that efficiency cannot be predicted solely by body size [42], [43], and other factors related to predator prey encounter rate need to be considered [44], [45]. The ability to survive during periods of extreme resource limitation affects the competitive capability of a species. In some zooplankton groups, starvation resistance has been related to organism body size [46], [47]. Lapesa [48] showed that the smallest of three studied *Brachionus* species was the least able to endure starvation.

The effect of slight morphological differentiation on the biotic dimensions of the niche of competing cryptic species has strong implications for fundamental problems in ecology such as limiting the similarity or the degree of ecological differentiation needed to promote coexistence. Previous studies have examined the differential susceptibility to predation and exploitative competition, including feeding strategies, of some species of the complex *B. plicatilis* [35], [36], [25]. However, no study has addressed this question by comparing two extremely morphologically similar species belonging to this complex: *B. plicatilis* and *B. manjavacas*. The assumption seems to have been that subtle morphological differences do not allow such ecological differentiation; however, this assumption needs to be evaluated, especially in the framework of coexisting cryptic species. In this study, we address the differential ecological response between *B. plicatilis* and *B. manjavacas*. We study the following ecological features known to be affected by body size: (1) vulnerability to copepod predation, as measured by the number of preyed upon neonates; (2) functional response, as measured by the clearance rates of both species using two different algae species as food resources; and (3) tolerance to starvation, as measured by growth and reproduction. As the differences are expected to be subtle, methodological attention was given to the statistical power of the data analysis.

## Material and Methods

### Rotifer species

We used two species of cyclical parthenogenetic rotifers belonging to the *B. plicatilis* cryptic species complex: *B. plicatilis* and *B. manjavacas*. The reproductive cycle of rotifers of the genus *Brachionus* begins with the hatching of asexual females from diapausing eggs. Females reproduce by ameiotic parthenogenesis for several generations, producing clones. Sexual reproduction is density-dependent and induced by a chemical cue [49], [50]. Then, asexual females begin producing sexual females that produce haploid oocytes that then develop into males if unfertilized or into diapausing eggs if fertilized.

### Media and culture conditions

The rotifers were fed two species of microalgae, which differ in size and mobility: *Tetraselmis suecica* (Prasinophyceae, motile, ellipsoidal, equivalent spherical diameter, ESD = 9 µm; provided by the Collection of Marine Microalgae of the Instituto de Ciencias Marinas de Andalucía, Cádiz, Spain) and *Nannochloris atomus* (Chlorophyceae, non-motile, spherical, ESD = 2.5 µm; strain CCAP 251/7; provided by the Collection of Algae and Protozoa of the Scottish Association of Marine Sciences, Oban, Scotland). The microalgae species were individually cultured at 20.0±0.1°C in an f/2 enriched saline water medium [51] at 10 g/L salinity under constant aeration and illumination (35 µmol quanta m^−2^ s^−1^). This salinity was selected because it is in the range of optimal values for the studied rotifer species in Salobrejo Lake [31]. Saline water was created with commercial sea salt (Instant Ocean®; Aquarium Systems). The microalgae were maintained in exponentially growing, semi-continuous cultures (dilution rate: 0.5 day^−1^) to provide food of constant quality during the experiments. Microalgae density was estimated by 750-nm wavelength light extinction using an absorption vs. density calibration curve. The equivalence to carbon content per microalgae cell was estimated using an elemental analyzer with thermal conductivity, EA 1108 CHNS-O (Fisons Instruments), using the flash combustion technique. Unless otherwise indicated, the rotifers were cultured under the same standard conditions of temperature, salinity and illumination as the microalgae.

### Rotifer isolation and species identification

The rotifer clones used in the experiments were established from diapausing egg hatchlings. Sediment containing these eggs was collected in June 2010 with a Van Veen grab (Eijelkamp Agrisearch Equipment) from the upper sediment layer of Salobrejo Lake (Eastern Spain, 38°54.765′N, 1°28.275′O). The sediment samples were stored in the dark at 4°C for 30 days to ensure the completion of the obligate period of dormancy of the diapausing *Brachionus* eggs [52]. Diapausing eggs of *B. plicatilis* and *B. manjavacas* were isolated from the sediment samples using a modified sucrose flotation technique [53]. The eggs were then individually transferred to 96-well plates (NuncTM) containing 150 µL of 10-g/L saline water and induced to hatch under the following conditions: 25.0±0.1°C and constant illumination (150–170 µmol quanta m^−2^ s^−1^). The eggs were checked every 24 h, and neonate females hatching from the eggs were isolated, fed with *T. suecica* (250,000 cells mL^−1^, ≈33 mg C L^−1^), and allowed to found clones by parthenogenetic proliferation.

Species identification of the clones was performed by polymerase chain reaction (PCR) and restriction fragment length polymorphism (RFLP) analysis of a fragment of the mitochondrial gene cytochrome c oxidase subunit I (COI) [33]. DNA was extracted from 5–7 females per clone using the HotSHOT method [54], and the mitochondrial COI fragment was amplified using PCR using the invertebrate universal primers LCO1490 and HCO2198 [55] as described in [56]. The RFLP analysis was performed with Kpn I and Pvu II endonucleases following Campillo et al. [33].

Stock cultures of 25 clones from each rotifer species were maintained separately under standard conditions. Prior to the experiments, multiclonal pre-experimental populations of *B. plicatilis* and *B. manjavacas* were established under different experimental conditions (see below) by mixing approximately 25 females of each of the 25 clones (approximately 1 female mL^−1^ of each clone). These populations were cultured for three generations to reduce maternal effects (e.g., [57]) and to acclimate the rotifers to the experimental conditions.

### Clearance rates

The feeding behavior of *B. plicatilis* and *B. manjavacas* was studied by measuring their clearance rates in short-term feeding experiments in monoalgal cultures of *T. suecica* and *N. atomus* following Ciros-Pérez et al. [43]. Four rotifer multiclonal pre-experimental populations were established (2 rotifer species×2 microalgae species). The rotifers were transferred from the pre-experimental cultures to the experimental food concentration 1 h before the experiments. For that purpose, these cultures were filtered through a 30 µm Nitex mesh, and the retained rotifers washed with saline water at 10 g/L to eliminate any remnants of algae. Afterwards, the rotifers were transferred to Petri dishes containing a culture medium with the experimental concentration of algae. The experiments were performed by pipetting 20 rotifers for *T. suecica* and 40 rotifers for *N. atomus* into Eppendorf® tubes with 1 mL algae culture at a concentration of 0.6 mg C/L. The tubes were kept for 1 hour in a centrifuge at a constant speed (6 rpm), at 20°C, and in darkness to avoid algal growth during the experiment. After 1 hour, the tubes were fixed with 20 µL of Lugol's solution. Ten replicates were performed for each rotifer-algae combination. Additionally, three tubes with *T. suecica* and three tubes with *N. atomus* without rotifers were used as controls and fixed immediately after inoculation with the algae. The experimental concentration of each algae species was 0.6 mg C/L, which corresponds to 3,140 cell/mL of *T. suecica* and 375,000 cell/mL of *N. atomus*. According to Ciros-Pérez et al. [43], this concentration of food is below the incipient limiting level (ILL) for both *T. suecica* and *N. atomus*. The clearance rate remains constant below the ILL [58], a critical food concentration from which the clearance rate exponentially decreases [59]. Below this level, filtration rates decrease linearly with decreasing food concentrations. However, to confirm that our experimental food concentration was below the ILL, three additional tubes for each rotifer-algae combination were prepared following the same procedure, except that the incubation was for 2 hours prior to fixation.

The algae were counted using an inverted Olympus® SZK10 microscope. A minimum of 800 cells were counted per sample to obtain a confidence interval of 7% [60]. The clearance rates were calculated following Peters [61]:
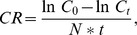
where 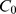 and 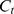 are the initial and final algae concentrations, *N* is the rotifer density, and *t* is the time in hours. For each microalgae species, the 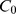 value was the average concentration of the three control tubes.

The data were independently analyzed for each algal species. To confirm whether the experimental algae concentration was under the ILL, a linear regression analysis (algae concentration vs. incubation time, i.e., 0, 1 or 2 h) was performed using R version 2.12.1 (R Foundation for Statistical Computing, 2010). Student's t test was used to test for differences between *B. plicatilis* and *B. manjavacas* in CR for each alga. The highest and lowest values for each species were excluded from the analysis. Additionally, to test the power of our analysis, the minimum detectable statistically significant difference given the observed experimental variance was computed; the CR value of one species remained fixed while the mean CR value of the other species was gradually increased, without modifying the variance, until the difference between the two groups was statistically significant. These analyses were performed using SPSS version 9.0 (SPSS Inc., Chicago, Illinois).

### Predation susceptibility

The relative susceptibility of *B. plicatilis* and *B. manjavacas* to predation by *Arctodiaptomus salinus* (Copepoda, Calanoida) was tested through differential predation experiments on the rotifer species. This copepod was selected as the predator because the adult stage feeds on small zooplankters including species of the *B. plicatilis* complex [36] and because *A. salinus* co-occurs with *B. plicatilis* and *B. manjavacas* (e.g., in Salobrejo Lake; [36]; Montero-Pau, personal communication); thus, this copepod is a potential predator of both species and might play an important role in the coexistence of these two cryptic species if the predation were differential.

Diapausing eggs of *A. salinus* were isolated using the same sucrose flotation technique from the same sediment samples from which both rotifer species were obtained. The copepod eggs were incubated under standard conditions until they hatched. The nauplii were individually isolated in 24-well plates (NuncTM) and maintained on a mixed diet of *T. suecica* and *N. atomus*. The medium was renewed every 5–7 days, and the copepods reached the adult stage in approximately 3–4 weeks.

For the predation experiments, we selected rotifer neonates as prey from pre-experimental multiclonal populations (see above). *A. salinus* prefers small prey [36]. Thus, by using neonates, predation is expected to be more efficient. We performed two predation experiments. In the first, we used only adult copepod females as predators, whereas we examined both sexes separately in the second to test for differential predation by adult females and males. The procedure in both experiments was the same. Adult copepods were individually placed in the wells of 24-well plates (NuncTM), with each well containing 1 mL of 10 g/L saline water without food. After 15–16 hours, 25 rotifer neonates were added per well. Both rotifer species were tested separately. Ten replicates plus three controls without copepods, to control for mortality due to other factors (i.e., the intrinsic mortality of rotifers), were completed for each rotifer species. The copepods and rotifers were incubated together for 24 hours. After that time, the copepods were removed, and the rotifers, including those in the controls, were fixed with Lugol's solution. The rotifers were counted under a Leica SZX2 stereomicroscope. The number of rotifers suffering predation was calculated as the difference between the initial and final counts in each well.

After checking for equal variances, the differences in the predation rate between the prey species in the first experiment were analyzed using Student's t test. The variances in the second experiment were not homogenous. Thus, a robust two-way ANOVA was applied to test for the effects of prey species and predator gender on predation. The power of our analysis and the minimum detectable statistically significant difference were computed. For each rotifer species, we randomly chose a surviving rotifer from one of the replicates and considered it as instead suffering predation, and then we statistically reanalyzed the simulated data. This process was repeated, accumulating randomly chosen individuals as suffering predation, until the difference between both species was significant. SPSS version 9.0 (SPSS Inc., Chicago, Illinois) was used to perform Student's t test, and R version 2.12.1 (R Foundation for Statistical Computing, 2010) was used to perform the robust two-way ANOVA.

### Tolerance to starvation

The response of *B. plicatilis* and *B. manjavacas* to different periods of starvation before the age of maturity was measured using a dynamic life table experiment. To establish the cohorts, aliquots of approximately 8 mL of pre-experimental multiclonal cultures were transferred into assay tubes and gently shaken to detach the eggs from the females [62]. The detached eggs harboring female embryos (the gender of an embryo can easily be distinguished by egg size) were removed and isolated on plates in 10-g/L saline water. These eggs usually hatch in less than 4–5 hours. The neonate females hatched within 2 h were individually transferred into 1 mL of saline water in the wells of 24-well plates (NuncTM). For each rotifer species, the neonates were divided into five cohorts containing 25 females each and assigned to one of five fasting times of 0, 6, 12, 18 or 24 hours (2 rotifer species×5 treatment of starvation = 10 cohorts). The species and starvation treatments were randomly distributed across the 24-well plates. After the corresponding hours of starvation, the females were fed *T. suecica* at a concentration of 100,000 cell/mL (≈13 mg C L^−1^). The females were followed and monitored every 24 hours until all died. Daily, the survival and number of offspring produced were recorded, and the female was transferred to a new well of a 24-well plate (NuncTM) containing 1 mL of fresh medium with 100,000 cell/mL of *T. suecica*.

The lifespan 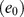, mean generation time 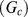, net reproduction rate 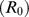, survival function (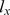, with 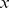 being age), and age-specific fecundity 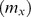 of both rotifer species under each starvation treatment were calculated. Comparisons among the survival curves were performed using two non-parametric tests: a Log-rank test and a Breslow test [63]. The former assumes equal importance of all observations, whereas the latter gives more weight to the initial part of the survival curve. These tests were performed using SPSS version 9.0 (SPSS Inc., Chicago, Illinois). In addition, potential intrinsic growth rates (i.e., the rate of increase that a population would have if no investment in sex occurred, 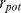) (Montero-Pau et al. unpublished manuscript) were obtained using the Euler-Lotka equation (e.g., [64]). The 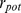 for a life-table experiment is obtained by exclusively considering the asexual fraction of the population. As births do not necessary occur at the moment of the observation, the estimated 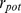 was improved by considering each time of observation 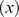 as the midpoint between this time and the next [65]. ANCOVA was performed to analyze the effects of species and starvation on 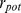,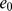, 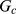 and 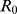. The ANCOVA results of 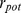 should be interpreted considering the intrinsic biases of the estimates of 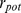 [65], [66]. However, these ANCOVA results were supported by the 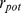 confidence intervals. These analyses were performed using R version 2.12.1 (R Foundation for Statistical Computing, 2010). The 95% confidence intervals of 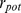 were obtained using bootstrap resampling [65] and corrected following the bias-corrected percentile method [67], [68]. The bootstrapping and its correction were implemented in R version 2.12.1 (R Foundation for Statistical Computing, 2010), and 10,000 randomizations were performed for each treatment and species.

### Niche overlap

Interspecific biotic niche overlap was estimated based on the studied features using the analytical approach of Geange et al. [69]. This method can account for multiple niche axes, each characterized by different data types, and computes a unified analysis of niche overlap. We used the clearance rates, susceptibility to predation, and starvation tolerance data sets to calculate the biotic niche overlap between *B. plicatilis* and *B. manjavacas* along the following eight axes: (1) Clearance rate for *T. suecica* (continuous data); (2) Clearance rate for *N. atomus* (continuous data); (3) Susceptibility to predation by copepod females (binary data); (4) Susceptibility to predation by copepod males (binary data); (5) Potential intrinsic growth rate after 6 h of starvation (continuous data); (6) Potential intrinsic growth rate after 12 h of starvation (continuous data); (7) Potential intrinsic growth rate after 18 h of starvation (continuous data); and (8) Potential intrinsic growth rate after 24 h of starvation (continuous data). Before analysis, the clearance rates were log-transformed, and the 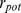 values were corrected by subtraction from the 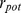 values obtained in the 0 h starvation treatment (see above) to remove the constant (starvation independent) interspecific effect. Niche overlap indexes (NO) were calculated for each dimension following Geange et al. [69]. Then, a single unified niche overlap index (Geange et al. [69]) was obtained by averaging the niche overlap over each different axis *t* as follows:
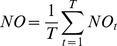
where *T* is the number of dimensions, and NO ranges from 0 (disjoint niches) to 1 (total niche overlap).

To assess the statistical niche differences between species, null model permutation tests were performed to test whether both the niche overlap along each axis and the mean niche overlap were significantly lower than expected by chance [69], [70]. Statistical null distributions (the distribution of the test statistic under the null hypothesis of no niche differentiation) were generated by calculating pseudo-values through randomly permuting species labels in the corresponding data set over 10,000 runs. The distribution of the average niche overlap for the null model was then computed. To correct for multiple comparisons, we performed a sequential Bonferroni adjustment [71].

The niche overlap calculations and associated null model tests were performed using R version 2.12.1 (R Foundation for Statistical Computing, 2010) using the source code provided as supporting information in Geange et al. [69].

## Results

### Functional response

**Figure 1:**
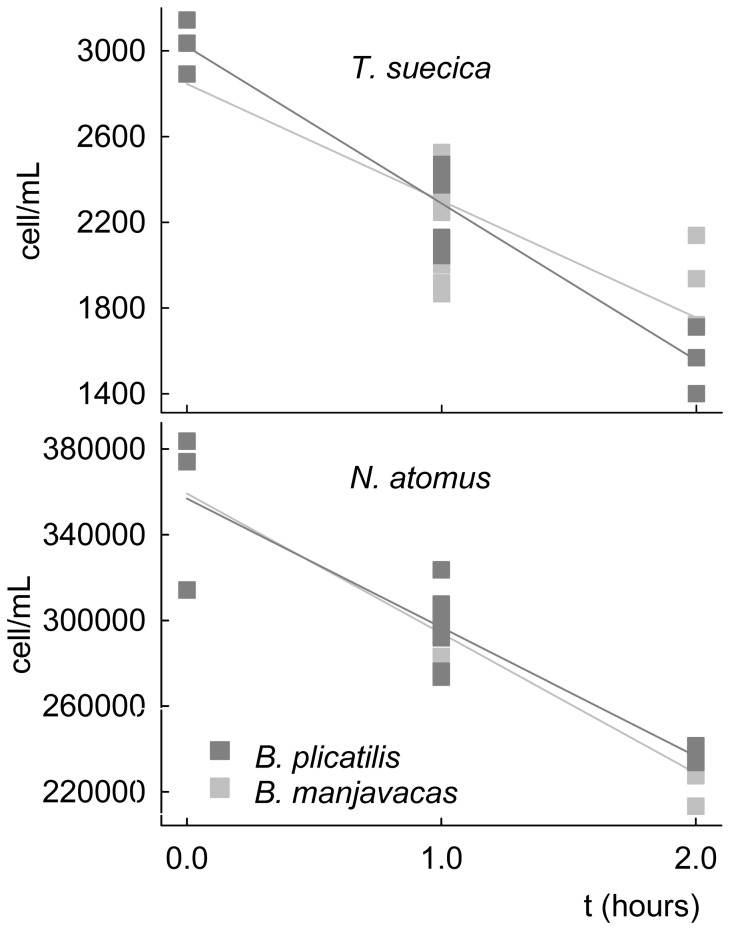
Concentration (cell/mL) of *T. suecica* and *N. atomus* for different incubation times with *B. plicatilis* and *B. manjavacas*.

The log concentration of both *T. suecica* and *N. atomus* decreased linearly (R^2^>0.68) with increasing incubation feeding time for both rotifer species (Fig. 1), indicating that the experimental food concentrations were below the incipient limiting level (ILL) [58], the threshold food concentration up to which clearance rates remain constant.

The averaged clearance rates for both rotifer species are shown in Table 1. When the rotifers were fed *T. suecica*, *B. plicatilis* presented a clearance rate that was on average 3.9% higher than that of *B. manjavacas*. In contrast, when they were fed *N. atomus*, *B. manjavacas* filtered 4.2% more than *B. plicatilis*. However, these differences were not statistically significant (Student's t test *P* = 0.72 for *T. suecica* and *P* = 0.24 for *N. atomus*). The power analysis demonstrated that, given our data variance, the difference between the average clearance rates of the two rotifer species would be need to be 30% to detect a statistically significant difference at the 5% significance level for rotifers fed *T. suecica*, whereas a 15% difference would be required when using *N. atomus* as food. Both *B. plicatilis* and *B. manjavacas* were three times more efficient when feeding on *T. suecica* than on *N. atomus*.

**Table 1:** Clearance rates (µL ind^−1^ h^−1^) of *B. plicatilis* and *B. manjavacas* feeding on the microalgae *T. suecica* and *N. atomus*.

Rotifer species	*T. suecica*	*N. atomus*
*B. plicatilis*	15.5±1.1 (11)	4.7±0.2 (8)
*B. manjavacas*	14.9±1.3 (11)	4.9±0.3 (10)

Values are the means ± SE (sample size in brackets). Estimations based on 1 h observations.

### Predation by copepods

**Figure 2:**
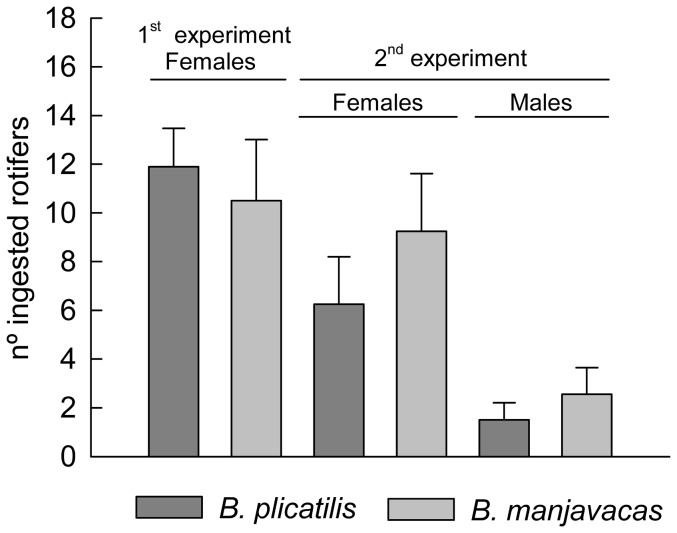
Average numbers of *B. plicatilis* and *B. manjavacas* consumed by *A. salinus* females and males in two predation experiments. Vertical bars are ± SE.

No rotifers died in the control replicates, so mortality was due to copepod predation. *A. salinus* females preyed 12% more on *B. plicatilis* than on *B. manjavacas* in the first experiment. In contrast, *B. manjavacas* was preyed upon on average 32% (female predators) and 41% (male predators) more than *B. plicatilis* in the second experiment. However, no statistically significant difference was found in either assay (Fig. 2; *P* = 0.642 and 0.287, for the first and second experiments, respectively). The results of the second experiment revealed a significant effect of copepod sex in the efficiency of predation (*P* = 0.003). The *A. salinus* females had four times higher predation efficiency than copepod males on both *B. plicatilis* and *B. manjavacas*. According to the power analysis, the difference between the predation efficacies of *A. salinus* on *B. plicatilis* and *B. manjavacas* must be at least 39% to be statistically significant at the 5% significance level in the first experiment (*P* = 0.045). At least a 49% difference between the predation efficiencies of *A. salinus* females and a 48% difference between the efficiencies of *A. salinus* males were needed in the second experiment (*P* = 0.049).

### Tolerance to starvation

**Figure 3:**
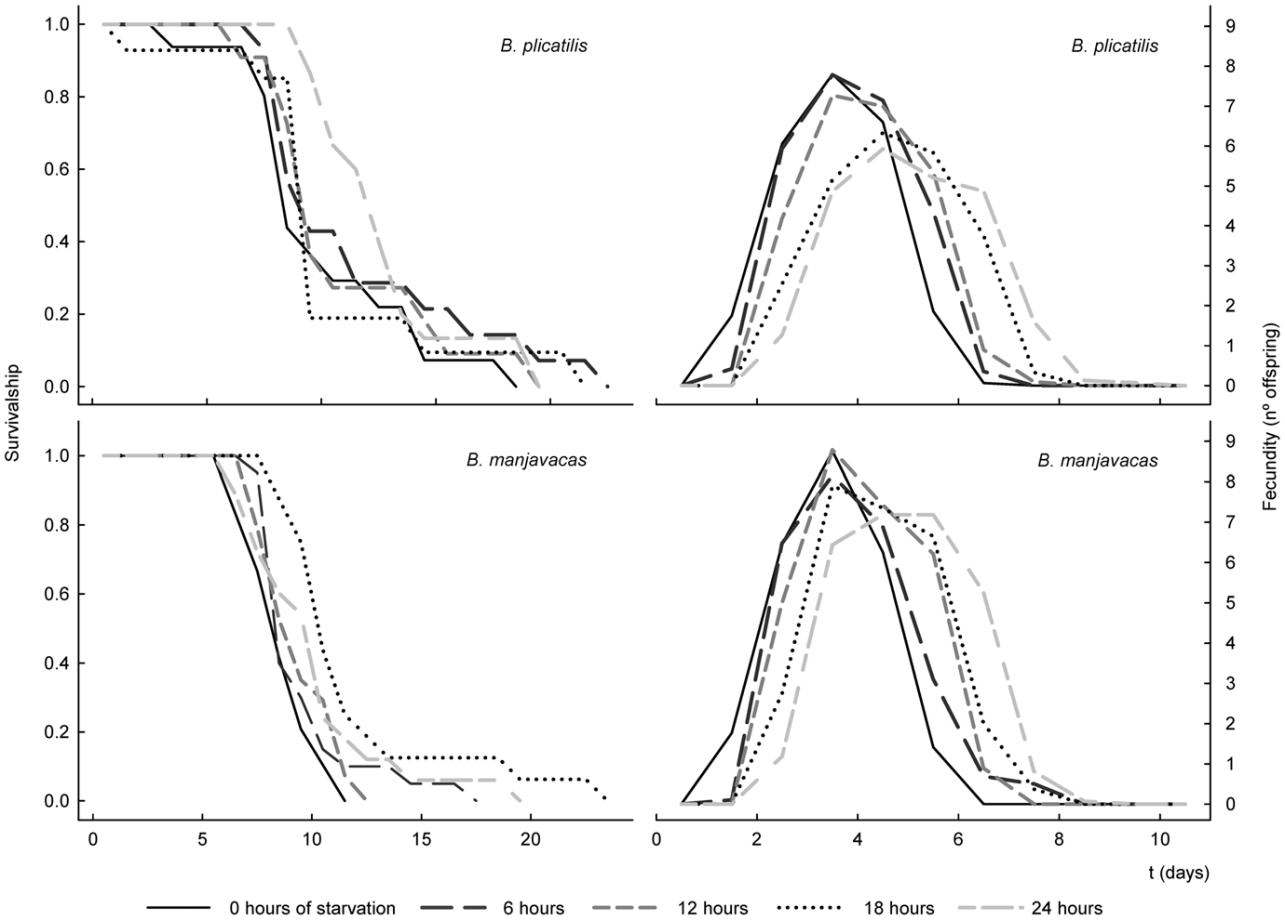
Survival and fecundity curves of *B. plicatilis* and *B. manjavacas* under different starvation times.

**Figure 4:**
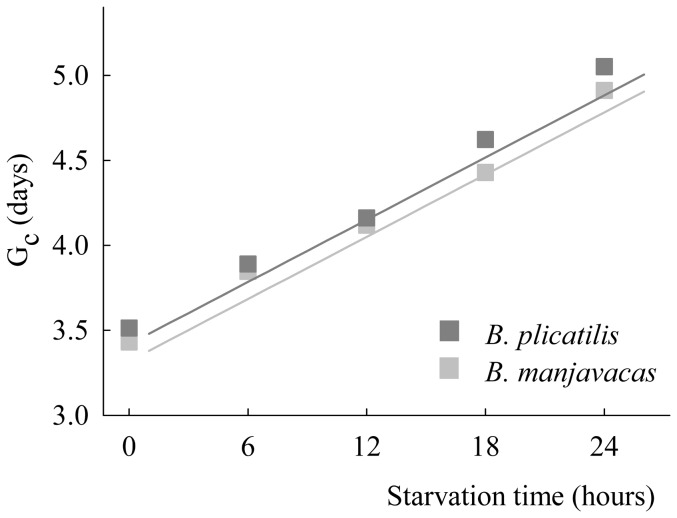
Mean generation times (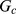) of *B. plicatilis* and *B. manjavacas* under different starvation times. Regression lines are shown.

Fig. 3 shows the 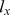 and 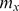 curves of *B. plicatilis* and *B. manjavacas* females under different starvation times. Both non-parametric tests (log-rank and Breslow) failed to find statistically significant differences between the survival functions when all of the survival functions of *B. plicatilis* and *B. manjavacas* were globally compared (*P* = 0.065 and 0.1, respectively). Moreover, no differences were found in survival when the data for each starvation level were compared between species (all *P*>0.12). The fecundity pattern of both rotifer species showed similar trends: maximum fecundity tended to decrease with starvation time, whereas the length of the reproductive period increased (Fig. 3). 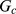 also tended to be positively related to increasing starvation period (Fig. 4) and was dependent on species, with *B. plicatilis* having slightly higher values than *B. manjavacas* (*P* = 0.036). However, the increasing effect of starvation on 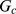 was similar in both species, with no significant differences between the slopes of the regression lines relating 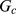 to starvation time (*P* = 0.369). The rotifer species did not show significantly different responses of 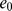 (*P* = 0.07) or 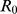 (*P* = 0.41) to starvation. Additionally, no significant differences were found in these parameters when both species were compared (*P* = 0.169 and 0.079, for 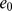 and 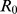, respectively).

**Figure 5:**
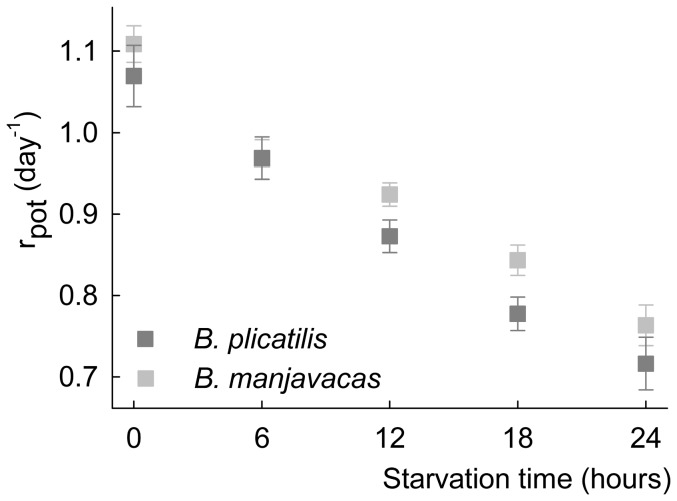
Response of the potential intrinsic growth rates (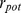) of *B. plicatilis* and *B. manjavacas* to starvation time following birth. Vertical bars are the lower and upper 95% confidence intervals.

The potential intrinsic growth rates for starved *B. plicatilis* and *B. manjavacas* females under the different starvation treatments are shown in Fig. 5. ANCOVA revealed that, regardless of the species, increasing starvation period led to decreasing 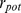 (*P*<0.001). In addition, statistical analysis indicated an effect of species on 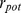 values (*P*<0.039); *B. manjavacas* had slightly higher values of 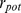 than *B. plicatilis* for all treatments. However, both species responded similarly to starvation time (i.e., equal slopes, *P*-value for species-treatment interaction = 0.275).

### Niche overlap

The niches of *B. plicatilis* and *B. manjavacas* were found to be very similar (mean niche overlap = 0.78, *P*-value = 0.162) as the overlap values for seven of the eight axes were high, and the analysis showed no significant differences between the two species (Table 2). Tolerance to 24 h fasting appeared to be a distinguishing factor between the species as it was the only axis indicating significantly different niches, but it did not remain significant after sequential Bonferroni correction. The most similar niches were associated with the axes related to predation by female and male copepods.

**Table 2:** Niche overlap indexes (NO) between *B. plicatilis* and *B. manjavacas* for the analyzed niche axes.

Niche Axis	NO	P-value*
CR_Ts_	0.798	0.450
CR_Na_	0.852	0.906
D_f_	0.954	0.166
D_m_	0.958	0.118
r_pot6_	0.755	0.345
r_pot12_	0.707	0.282
r_pot18_	0.673	0.210
r_pot24_	0.546	**0.033**

*CR_Ts_* and *CR_Na_*, clearance rates for *T. suecica* and *N. atomus*; *D_f_* and *D_m_*, predation by female and male copepods; *r_pot6_*, *r_pot12_*, *r_pot18_* and *r_pot24_*, potential intrinsic growth rate after 6, 12, 18 and 24 hours of starvation, respectively.

* Axis with statistically different niches, significant at *P*<0.05 as identified by a null model test, are indicated in bold. No value remained significant after sequential Bonferroni correction.

## Discussion

Difference in body size is an important mean by which species avoid direct overlap in resource use and can have important effects on vulnerability to predation [72], [73]. Size influences interspecific ecological interactions and has been proven to be an important feature related to the biotic dimensions of the niches of cryptic species, for example, as in the case of bats or amphipods [74], [75]. The primary objective of this paper was to examine the extent to which a subtle body size differentiation could cause differences in the biotic dimensions of the niche. In some species of the *B. plicatilis* complex, body size differences ranging from 23 to 50% have been shown to be associated with ecological differentiation [36], [35], [59], [76]. However, in this study, no major differences in vulnerability to predation, food particle size preference, or starvation tolerance have been found between *B. plicatilis* and *B. manjavacas*, which are virtually identical in morphology but differ by 6% in body size.

### Functional response

Body size is considered to be an important factor in determining trophic niche for filter-feeding organisms because the widths of their mouths limit the size of particles that they can ingest [77], [38]. In this study, we used two microalgae, *T. suecica* and *N. atomus*, which include a significant portion of the particle size preference range of *Brachionus* species [40], [78]. Our results demonstrated that the clearance rates for both rotifer species are equal despite their difference in body size. The values reported here are within the range described by other authors [39], [59], [43] for *Brachionus* species. *T. suecica* was filtered three times faster than *N. atomus*, which is smaller and immobile. These data are in agreement with the optimal prey size reported for *Brachionus* species [59], [79]. The clearance efficiencies of *B. plicatilis* for *T. suecica* were lower than recorded in previous studies [43]. This discrepancy might be because we used rotifer neonate females in our experiments, whereas female age was not controlled in Ciros-Pérez et al. [43]. Neonates are smaller than adults, and because the size of the particles that they can ingest is limited by body size, their algae filtration rates are expected to be lower.

The body size difference between *B. plicatilis* and *B. manjavacas* does not appear to be sufficient to affect their clearance rates. Moreover, assuming Tilman's model [80] and assuming that a stable equilibrium point exists in the use of the remarkably different resources tested here, our estimation of the clearance rates implies that the resource supply should be in a sector representing less than 1.5% of the resource space. Thus, niche partitioning by differential use of these microalgae seems highly unlikely, contrary to what has been observed in other species of the rotifer complex whose differences in body size are greater (*B. plicatilis*, *B. ibericus* and *B. rotundiformis* sizes ranging from 23 to 50%) [43], so that their coexistence of could be explained by differential clearance rates. Our conclusion of lack of differential resource use between *B. manjavacas* and *B. plicatilis* was supported by results from niche overlap analysis.

There are factors in addition to body size that affect prey selection in filter-feeding organisms and could also shape the trophic niche. Selectivity has been linked to algal characteristics such as cell surface [81], [82], physiological conditions [83] and motility [84]. Actively moving prey – microalgae in this case – may increase their encounter rates with predators [44]. The presence of cilia provides mobility to *T. suecica*, which, in addition to its larger size, may account for the higher grazing rates on these microalgae observed in our experiments for *B. plicatilis* and *B. manjavacas*.

### Tolerance to starvation

In aquatic systems, resource quantity and quality can vary drastically over short periods of time, and episodes of severe resource scarcity are expected. The time scale of these phenomena may vary from hours, due to daily vertical migration [85], to days or weeks during seasonal change [86], [87]. Accordingly, the ability to withstand starvation is considered an important element in species persistence [88], and this ability may affect the competitive outcome between zooplankton species [89], [46], [47], [90], [88], [59]. We found that the effect of food limitation on survival was similar in *B. plicatilis* and *B. manjavacas*. In accordance with previous studies on the response of *B. plicatilis* to starvation [91], *B. plicatilis* and *B. manjavacas* both altered their fecundity schedule under food-limited conditions. Starvation seems to cause a general delay in age-related reproductive traits in both species in our study: longer generation times, older age at maturity, and longer reproductive periods were recorded in our experiments. In contrast, the life expectancies and net reproduction rates of the two studied species were not affected by food limitation. The generation time and the potential growth rate are correlated. Thus, consistent with the effect of starvation on the generation time, the potential growth rate decreased linearly with increasing starvation time. *B. manjavacas* had slightly but consistently higher 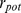 than *B. plicatilis* in all of the starvation treatments, although the differences were not statistically significant. Interestingly, both species are able to maintain positive growth rates after starving for one day, which is approximately 9% of their life expectancy. Starved newborn female rotifers are able to survive several days using egg reserves as their only source of energy [92], [91], [93], [94]. However, tolerance to this longer starvation time was the only axis suggesting niche segregation between the two species.

### Predation by copepods

Most of the rotifers species co-occurring with predatory copepods have been described as prey of copepods [95], [96]. In addition to prey size, vulnerability to predation also depends on morphological and behavioral features that could protect individuals from being successfully attacked [95], [97], [98]. In contrast with other rotifers, the *Brachionus* species studied here do not exhibit conspicuous features to avoid predation by copepods, except for a hard, chitinous lorica and a “dead-man” behavioral response to attacks [25]. Because *Brachionus* species are morphologically very similar, their size becomes relevant, with the highest susceptibility to predation being associated with the smallest sized species of the complex [36], [35], [25]. Therefore, the morphological and size similarity between *B. plicatilis* and *B. manjavacas* provides an explanation for their very similar vulnerability to predation by both male and female copepods, the tested axes that seemed to contribute most to their high interspecific niche overlap. We recorded predation rates by *A. salinus* in the range of those obtained by Lapesa et al. [36] when they studied predation of *A. salinus* on *B. plicatilis*. Our finding of sex-dependent predation efficiency in *A. salinus* is also consistent with the experimental data obtained by Ciros-Pérez et al. [35] using the copepod *Diacyclops bicuspidatus odessanus* as predator. The higher efficiency of female copepods may be due to their larger size. Prey handling time is expected to be negatively related to predator-prey size ratio, and a higher handling time involves a lower predation efficiency [99]. Interestingly, although the difference was not statistically significant, the females and males of *A. salinus* preyed slightly more on the smaller species, *B. manjavacas*.

### Ecological similarity

This study did not detect evidence that the cryptic species *B. plicatilis* and *B. manjavacas* differentiate their biotic niches by having different clearance rates, susceptibility to predation, or starvation tolerance. Three concerns could arise from this conclusion. (1) Generally, no test can rigorously demonstrate a lack of difference between data sets because that implies the acceptance of the null hypothesis. Thus, knowing the statistical power of the tests is critical to assessing the plausibility of the absence of differences. In our study, low statistical power might affect the predation experiment results, although we were able to detect significant differences for one factor (predator sex); this was likely because, although the sample size was small, our design controlled for prey age in the experiments, thus reducing the variability in intraspecific prey size (error variance). Overall, this suggests that even if statistical significance could be achieved by increasing the sample size, the differences would be minor and of low ecological significance. (2) Another consideration is whether phenotypic plasticity causing morphological divergence could contribute to the evolutionary differentiation of the biotic niche of these species, because of a differential response of their morphology to an environmental factor. However, evidence suggests that the morphology of *Brachionus* species either respond similarly or do not respond to changes in environmental factors, at least for temperature and salinity [100]. Moreover, these species inhabit a spatially homogenous environment, so that individuals should experience similar developmental environments. In addition, we did not notice indications of character displacement when the species were grown together. (3) A third concern is whether unanalyzed niche axes could promote niche differentiation. We intentionally limited our study to those biotic axes related to resource use and predation vulnerability. However, efficiency in resource use might be dependent on physical environmental factors. Our hypothesis is that this dependence does occur in relation to salinity and temperature, which are critical mediating their long-term coexistence (see below).

The evidence for ecological similitude between the two cryptic species studied here is in agreement with the phylogenetic limiting similarity hypothesis, which predicts that phylogenetically closely related species are likely to possess ecological similarities [101], [5], [102], [103]. This hypothesis also assumes that a higher ecological similitude is likely to result in more frequent competitive exclusion. However, *B. plicatilis* and *B. manjavacas* co-occur in many lakes along the Iberian Peninsula, and their occurrence in the region has been traced back to the Pleistocene [27], [28]; thus, their coexistence is unlikely to be transient. The discovery of cryptic species co-occurring in the same habitat, particularly if the habitat is spatially homogeneous, raises new questions in explaining the coexistence of these species. Moreover, it challenges the limit of ecological differentiation needed to promote coexistence and favors the description of novel coexistence mechanisms not based on niche partitioning [11], [104].

Differential adaptation to abiotic factors, such as water salinity and temperature, is known to affect the coexistence of the *B. plicatilis* species complex [31], [105], [106]. For example, laboratory experiments show *B. plicatilis* grows better at lower salinities than *B. manjavacas*, although the salinity tolerance ranges of these two species largely overlap [31]. Because of this evidence and the abundances recorded in the field, it has been proposed that their coexistence could be mediated by salinity fluctuations providing differential advantages to the two species in turn [31]. However, mechanisms explaining stable coexistence that are not based on niche differentiation are also likely to be acting on the coexistence of *B. plicatilis* and *B. manjavacas*, as well as the other species belonging to the cryptic complex. As noted using modeling by Montero-Pau and Serra (2011), density-dependent investment in sex and diapause is able to mediate the stable coexistence of facultative sexuals with identical niches if the response to density is to some extent species-specific. In the genus *Brachionus*, sexual reproduction is density-dependent, and some level of differentiation in the chemical signal that induces sex seems to exist in sympatric populations of the *B. plicatilis* complex, including *B. plicatilis* and *B. manjavacas* [107]. Different patterns of sexual investment have already been described in some populations of the *B. plicatilis* species complex [108], [109]. Sexual patterns of *B. plicatilis* and *B. manjavacas* should be studied under different environmental conditions to explore whether a possible differential investment could act as an explanatory mechanism of their coexistence.

Despite only minor differences in morphology, closely related sympatric species can display different preferences for abiotic conditions, especially in relation to factors for which the adaptation to specific ranges is based on a physiological mechanism and only loosely related to morphology. If *B. plicatilis* and *B. manjavacas* differentially respond to abiotic factors so that those factors drive the outcome of competition, fluctuations in the abiotic environment could facilitate their coexistence. We propose as a hypothesis to be tested that the advantages that fluctuating salinity provides to each species are mediated not only through differential effects of salinity on population growth rate but also through differences in sex and diapause patterns.
